# Case Report: Hypocomplementemic urticarial vasculitis syndrome in a pediatric patient with complement factor 1 deficiency

**DOI:** 10.3389/fped.2024.1448094

**Published:** 2024-09-23

**Authors:** Sallie Lin, Dina Kafisheh, Melissa E. Elder

**Affiliations:** ^1^ Department of Pediatrics, University of Florida, Gainesville, FL, United States; ^2^ Department of Pediatrics, Division of Allergy, Immunology and Rheumatology, University of Florida, Gainesville, FL, United States

**Keywords:** urticarial vasculitis, HUVS, complement deficiency, CF1, pneumococcal infection, c1q antibody

## Abstract

Urticarial vasculitis (UV) is a type III hypersensitivity reaction, characterized by immune complex deposition in small vessels leading to complement activation. Hypocomplementemic urticarial vasculitis syndrome (HUVS) represents the most severe form of UV, manifesting as chronic and recurrent urticarial skin lesions with leukocytoclastic vasculitis on histology, hypocomplementemia, anti-C1q antibodies, and systemic organ involvement. This case study focuses on an adolescent who initially presented with invasive pneumococcal infection and was later diagnosed with two rare disorders: HUVS and coexisting complement factor 1 (CF1) deficiency by genotyping. The role of CF1 deficiency in the development of HUVS in this patient is uncertain but has not previously been described.

## Introduction

Urticarial vasculitis (UV) is characterized by antigen–antibody complex deposition in small vessels, predominantly postcapillary venules, leading to complement activation (1, 2). UV clinically manifests as intermittent urticaria (1) and is diagnosed by the presence of leukocytoclastic vasculitis (LCV) on biopsy, including “leukocytoclastic reaction, vessel wall destruction, and deposits of fibrinogen” (1).

UV ranges on a spectrum from mild to severe: normocomplementemic urticarial vasculitis (NUV), hypocomplementemic urticarial vasculitis (HUV), and hypocomplementemic urticarial vasculitis syndrome (HUVS) (2, 3). In contrast to NUV and HUV, which are limited to skin manifestations occurring often for less than 6 months, the diagnosis of HUVS includes systemic organ involvement, which may be variable over time, and positive anti-C1q antibodies in the serum (1, 3). The following Case Report describes an adolescent male whose presentation of pneumococcal bacteremia and pneumonia later led to a diagnosis of HUVS based on a required persistent leukocytoclastic rash, C1q antibody positivity, hypocomplementemia, and frequent arthralgias without criteria for systemic lupus erythematosus (SLE). Subsequent genotyping confirmed complement factor 1 (CF1) deficiency as the likely major risk factor for his invasive pneumococcal infection and possibly the development of his HUVS. Prompt recognition of hypocomplementemic etiologies is important in allowing medics to provide optimal medical care and prevent life-threatening infections, of which pneumococcus is the most common.

## Case

The patient is a 14-year-old male who was admitted to the hospital for respiratory distress, in the setting of pneumonia and pleural effusion. His past medical history was significant for parental reports of waxing and waning urticarial and purpura rash over bilateral lower extremities sometimes extending to the trunk and upper extremities starting 7 years previously at 7 years of age, in association with intermittent knee and ankle arthralgias and uncertain arthritis, but without any serious infections reported. Per parent, he was previously treated with multiple steroid courses to ameliorate his chronic rash and joint complaints. Family reported the patient as being up to date on vaccines before emigrating from Central America, but no supporting documents were available.

**Figure 1:**
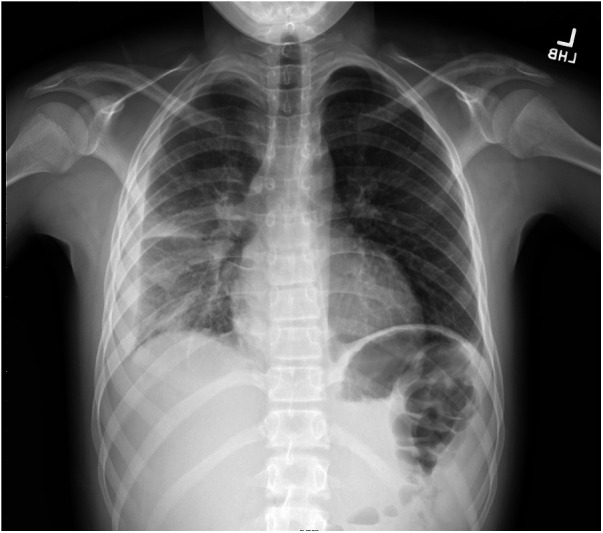
Right-sided pleural effusion and pneumonia.

**Figure 2:**
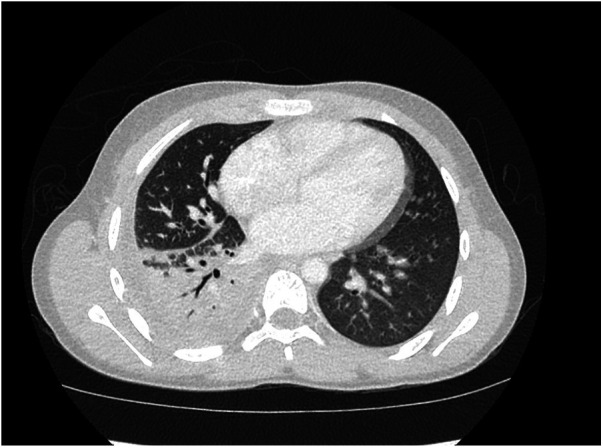
Pneumonia with opacification in the right lower lobe and right upper lobe, with a small loculated right parapneumonic effusion.

In the emergency department, a physical exam was notable for tachypnea, tachycardia, right-sided chest pain, and decreased breath sounds in the right lobes. No rash was noted initially but quickly developed over extremities during hospitalization. Symptoms leading to presentation included fever, fatigue, emesis, and increased breathing effort. Laboratory values were notable for lactate (4.59 mmol/L), blood urea nitrogen (BUN) (28 mg/dl), creatinine (1.18 mg/dl), and procalcitonin (5.52 ng/ml). He received intravenous (IV) fluids for the treatment of acute kidney injury, and IV antibiotics were started due to concerns about sepsis. He was admitted for respiratory support on a high-flow nasal cannula and antibiotics. Computed tomography confirmed right lower and upper lobe pneumonia, with a small loculated parapneumonic effusion (Figures 1, 2). He was initially treated with broad-spectrum antibiotics, including vancomycin, ceftriaxone, and azithromycin. Blood cultures were positive for pan-susceptible *Streptococcus pneumoniae*, and antibiotics were restricted to IV ampicillin. With clinical improvement and the resolution of pneumococcal sepsis and pneumonia, he was discharged home to complete a course of oral amoxicillin. No immunoglobulins, vaccine antibody titers, or complements were checked during hospitalization.

**Figure 3:**
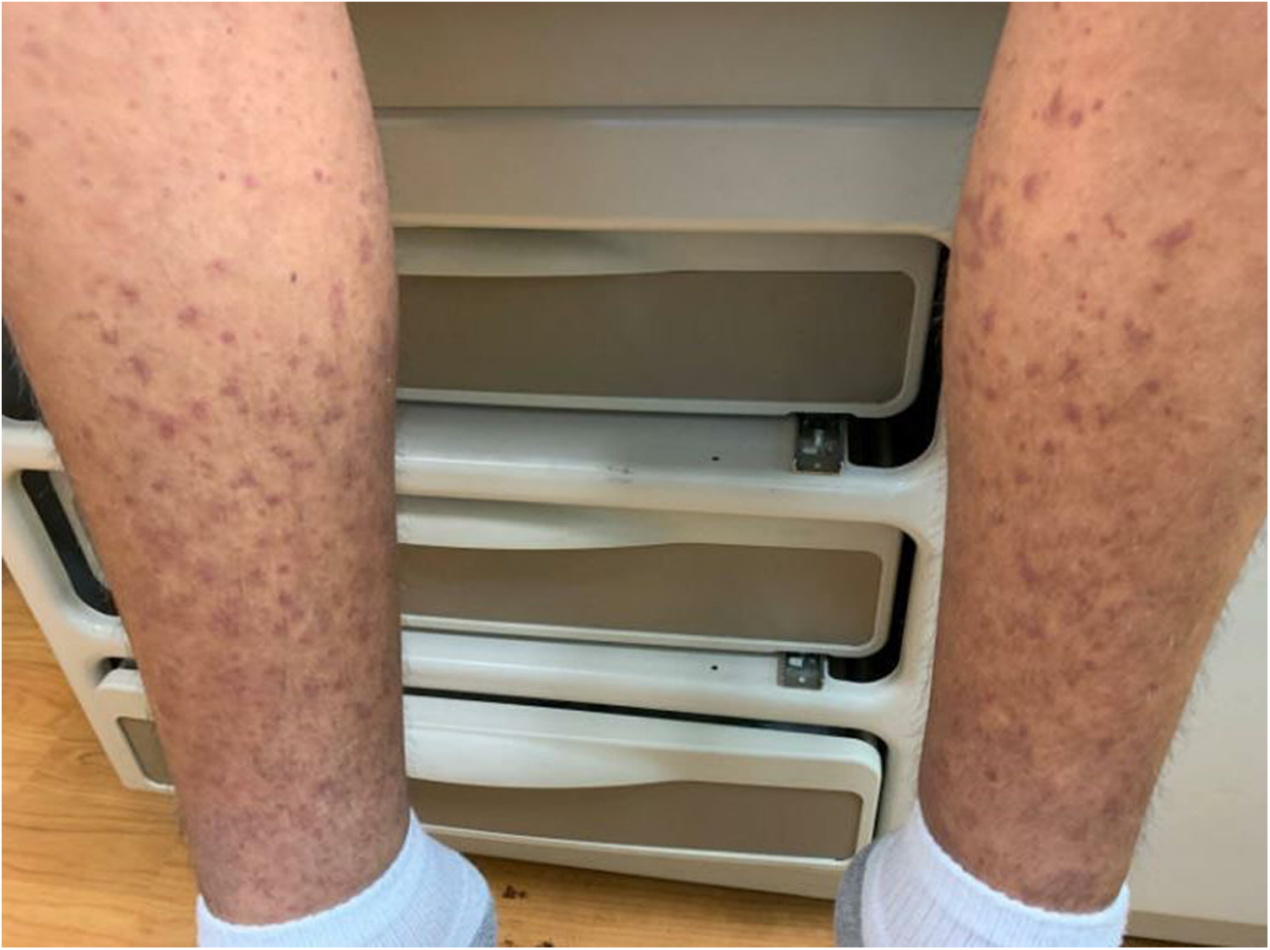
Rash on the lower extremities.

**Figure 4:**
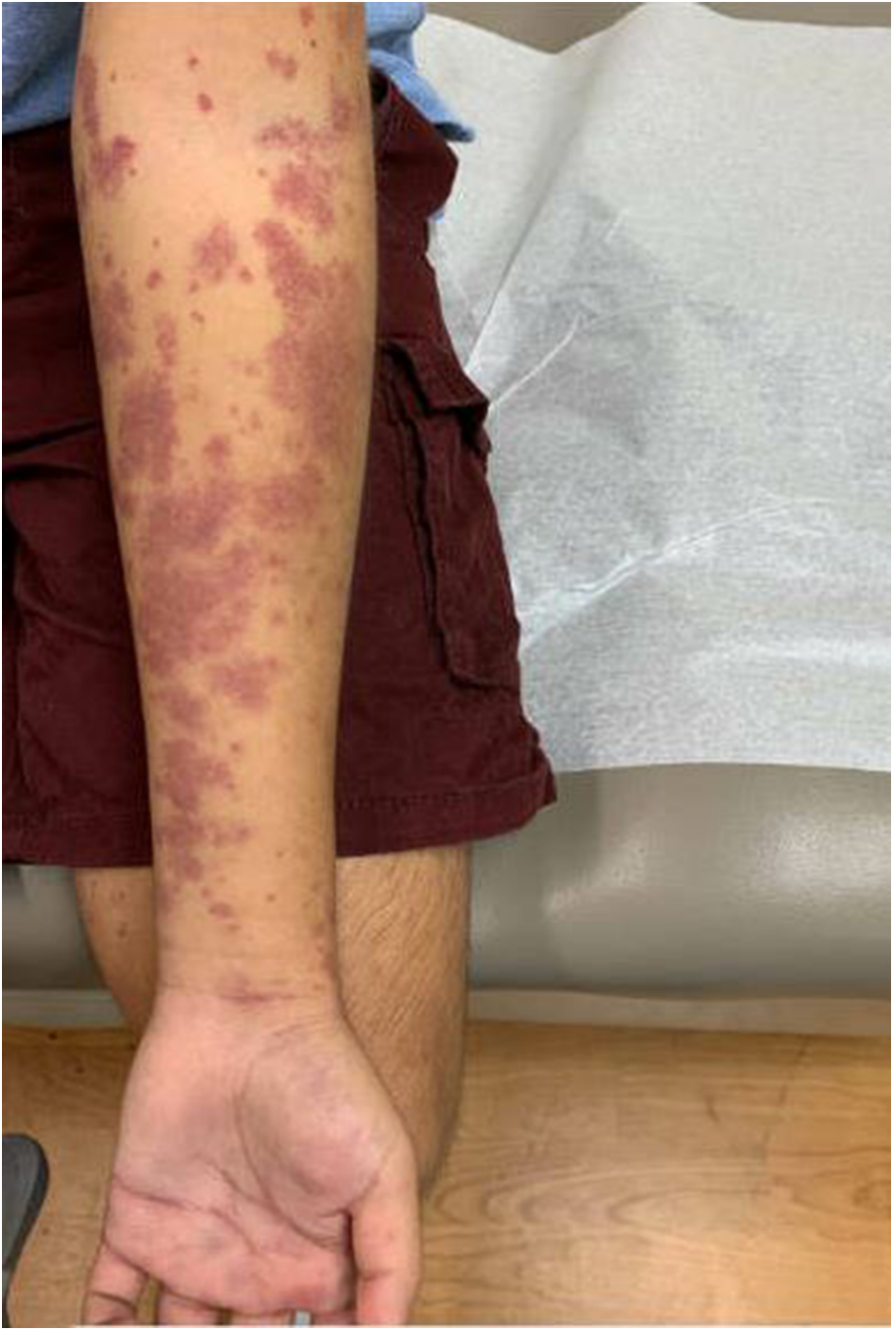
Rash on an upper extremity.

After discharge, the patient presented to a primary care clinic for a worsening vasculitic rash. Like the patient's reports, the rash was pruritic and associated with lower extremity swelling (Figure 3), extending to the upper extremities and trunk (Figure 4). As a result, he was referred to the Immunology Clinic 2 months later for further evaluation. IgG, IgA, and IgM were normal. IgE was elevated at 1,350** **kU/L. The C3 level was low at 18 mg/dl. The C4 level was normal at 20 mg/dl. CH50 was low at 27.1 U/ml. In addition, the complement C1q level was low at 60 μg/ml. Anti-C1q IgG was positive. Rheumatoid factor was 32 IU/ml. An antinuclear antibody (ANA) test was negative. C1 esterase inhibitor was normal at 31 mg/dl. Myeloperoxidase and proteinase 3 antibodies were negative.

Biopsy from right arm purpura demonstrated inflammatory cell infiltrate consisting of lymphocytes, histiocytes, neutrophils, and nuclear dust with minimal fibrin in the small blood vessel walls, consistent with LCV (Supplementary Figures 1, 2). In addition, an Invitae Innate Error of Immunity and Cytopenia panel was sent and revealed two pathogenic variants in the *CFI* gene, associated with autosomal recessive CF1 deficiency with a risk for recurrent pneumococcal infections.

Further evaluation showed that *Haemophilus influenzae* B (Hib) IgG and *Neisseria meningitidis* IgG titers were not protective. Despite documented pneumococcal disease and purported pneumococcal (type unknown) vaccination 1 month before hospitalization, his pneumococcal antibody titers were protective against only 5 of 14 serotypes at a level of >1.3 μg/ml 3 months after infection. The number of protective antibody titers increased to 8 of 14 serotypes after the administration of a first pneumococcal polysaccharide vaccine, and then to 13 of 14 serotypes after a second pneumococcal polysaccharide vaccine was given. Cardiac screening noted a normal sinus rhythm on electrocardiogram (EKG) and normal echocardiogram. B-type natriuretic peptide (BNP) was normal at 6 pg/ml. Uveitis or episcleritis were not present on ophthalmic exam. Renal studies, including urinalysis, creatinine, and BUN, were normal. As symptoms were limited to pruritus, joint pain, and edema, antihistamines and non-steroidal anti-inflammatory drugs (NSAIDs) as needed were recommended for supportive care. Given the low C3 and CH50, the patient was administered pneumococcal polysaccharide, meningococcal, and Hib vaccines and started on lifelong amoxicillin prophylaxis.

## Discussion

HUVS is rare, with an annual incidence of 0.5–0.7 cases per 100,000 people and a predominance in adult women, although it has been reported in children (1, 4). HUVS, also known as McDuffie syndrome, may be associated with autoimmune disorders such as SLE, complicating diagnosis (1–3, 5) The pathophysiology is characterized by chronic urticarial rash with LCV on histology, hypocomplementemia, decreased C1q with positive anti-C1q antibodies, and systemic organ involvement (1, 3, 4).

Recurrent urticarial rash for more than 6 months is the dominant clinical manifestation and is required for diagnosis, as are low circulating complement components (1, 3, 4). Arthralgia and arthritis are the most common systemic presentation of HUVS, occurring in up to 90% or more of cases (1). At least 20%–50% of patients will experience renal, pulmonary, ophthalmic, or gastrointestinal involvement but these are not absolutely required for a diagnosis of HUVS (1, 3). The risk of pyogenic infections is increased in HUVS patients, likely from hypocomplementemia (1). Of note, constitutional symptoms of fever and malaise are rare (3). In addition, cardiac involvement, such as pericarditis or valvular involvement, is rare but can be life threatening (3).

The diagnosis of HUVS includes two major criteria and at least two minor criteria as defined by the Schwartz Criteria (4, 6): major criteria (both required)—(1) recurrent urticaria of >6 months in duration and (2) hypocomplementemia; minor criteria (at least two positive)—(1) venulitis on dermal biopsy, (2) arthralgias or arthritis, (3) glomerulonephritis, (4) uveitis or episcleritis, (5) recurrent abdominal pain, and (6) a positive C1q precipitin test by immunodiffusion with a suppressed C1q level (1, 2, 6).

In this Case Report, the patient met the Schwartz criteria for HUVS. With symptoms starting 7 years previously, he had >6 months of recurrent urticaria with a leukocytoclastic rash, including during a 4-month stay in the USA and intermittently for the subsequent 18 months. Lab work confirmed hypocomplementemia with low classical complement proteins, including low C1q and positive anti-C1q antibody on repeated testing over time. A skin biopsy of the rash demonstrated leukocytoclastic vasculitis involving all small vessels. Urticarial flares were accompanied by arthralgias but not frank arthritis. He did not have cardiac, gastrointestinal, ocular, or renal manifestations. A previous rash, possible intermittent arthritis per parent, and flares of edema provided important clinical clues to the diagnosis of HUVS in this case. His gender and age at diagnosis are unusual but have been reported previously.

The diagnoses of pneumococcal bacteremia and pneumonia in a purportedly previously immunized adolescent were concerning with regards to an underlying immunodeficiency, as children less than 2 years old, adults 65 years and older, and unvaccinated adolescents and adults are generally considered the most susceptible to severe *Streptococcus pneumoniae* infections (7). Unfortunately, we do not have his vaccine antibody titers while he was hospitalized with pneumococcal sepsis. Genotyping to rule out an inherited primary immunodeficiency confirmed the patient did indeed have a primary immunodeficiency disorder due to CF1 deficiency. He is on amoxicillin prophylaxis for life as a result.

In a few cases of HUVS, mutations in *DNASE1L*3 have been reported in the medical literature (8). In our case, genetic studies found two pathogenic variants in the *CFI* gene, associated with autosomal recessive CF1 deficiency with a risk of recurrent bacterial infections, often severe pneumococcal disease (9, 10) and autosomal dominant atypical hemolytic uremic syndrome (HUS). It is unclear what role his CF1 deficiency played in the etiology of his HUVS but it certainly predisposed him to pneumococcal sepsis and pneumonia (11). The presence of two rare disorders in this patient that may have increased his risk of pneumococcal disease is even more unusual, with no other reported cases in the literature.

HUVS does not have an established standard treatment, as it may be affected by the presence of other autoimmune disorders, unlike in this case. Therapy may include corticosteroids alone or immunomodulatory or immunosuppressive interventions as indicated depending on the severity of the rash and organ systems involved (2, 3). As symptoms were limited to pruritus and joint pain in this case at presentation, supportive care with antihistamines and NSAIDs as needed was effective, in addition to amoxicillin prophylaxis and the administration of pneumococcal, Hib, and meningococcal vaccines.

## Conclusion

In conclusion, pneumococcal sepsis in a purportedly previously immunized adolescent is concerning with regard to an underlying primary immunodeficiency. The patient was diagnosed with CF1 deficiency by genetic testing and is on lifelong antibiotic prophylaxis. The presence of an additional persistent waxing and waning urticarial vasculitic rash for over 7 years complicated the diagnosis and treatment, and an evaluation supported a second rare diagnosis of HUVS. Moreover, coexisting HUVS and CF1 deficiency increase his risk for developing SLE or other autoimmune diseases over time even further. Any role of his primary immunodeficiency in the etiology of his HUVS is unclear. Understanding of the presentation of HUVS as well as CF1 deficiency is crucial for the prompt evaluation and correct treatment of children and adolescents with invasive pneumococcal disease.

## Data Availability

The original contributions presented in the study are included in the article/Supplementary Material; further inquiries can be directed to the corresponding author.

## Supplementary material

Supplementary Figure 1 Biopsy demonstrating leukocytoclastic vasculitis (200×).

Supplementary Figure 2 Biopsy demonstrating leukocytoclastic vasculitis (300×).

## References

[1] Buck A Christensen J McCarty M. Hypocomplementemic urticarial vasculitis syndrome: a case report and literature review. J Clin Aesthet Dermatol. (2012) 5(1):36. PMC3277093 22328958

[2] Kolkhir P Grakhova M Bonnekoh H Krause K Maurer M. Treatment of urticarial vasculitis: a systematic review. J Allergy Clin Immunol. (2019) 143(2):458–66. 10.1016/j.jaci.2018.09.007 30268388

[3] Vallianou K Skalioti C Liapis G Boletis JN Marinaki S. A case report of hypocomplementemic urticarial vasculitis presenting with membranoproliferative glomerulonephritis. BMC Nephrol. (2020) 21(1):351. 10.1186/s12882-020-02001-6 32811472 PMC7433181

[4] Kessarwani V Phachu D Trivedi R. Hypocomplementemic urticarial vasculitis syndrome or systemic lupus erythematosus in evolution. Cureus. (2022) 14(3):e23429. 10.7759/cureus.23429 35481300 PMC9033644

[5] Tsai CC Lin CH Wang YC Chang FY. Acute respiratory distress syndrome in a man with Epstein-Barr virus infection-induced hypocomplementemic urticarial vasculitis. J Formos Med Assoc. (2018) 117(5):452–3. 10.1016/j.jfma.2018.01.022 29458992

[6] Schwartz HR McDuffie FC Black LF Schroeter AL Conn DL. Hypocomplementemic urticarial vasculitis: association with chronic obstructive pulmonary disease. Mayo Clin Proc. (1982) 57(4):231–8. 7040825

[7] Tan TQ. Pediatric invasive pneumococcal disease in the United States in the era of pneumococcal conjugate vaccines. Clin Microbiol Rev. (2012) 25(3):409–19. 10.1128/CMR.00018-12 22763632 PMC3416489

[8] Özçakar ZB Foster J Diaz-Horta O Kasapcopur O Fan YS Yalçınkaya F DNASE1L3 mutations in hypocomplementemic urticarial vasculitis syndrome. Arthritis Rheum. (2013) 65(8):2183–9. 10.1002/art.38010 23666765

[9] Arkwright PD. “Primary immunodeficiencies of complement”. In: Bernstein JA, editor. Primary and Secondary Immunodeficiency. Cham: Springer (2021). p. 313–30.

[10] Wisner E Kamireddy S Prasad P Wall L. P197 macrophage activation syndrome as the initial presentation of C1q deficiency. Ann Allergy Asthma Immunol. (2016) 117(5):S80–1. 10.1016/j.anai.2016.09.208

[11] Alba-Domínguez M López-Lera A Garrido S Nozal P González-Granado I Melero J Complement factor I deficiency: a not so rare immune defect. Characterization of new mutations and the first large gene deletion. Orphanet J Rare Dis. (2012) 7(42):1–8. 10.1186/1750-1172-7.42 22710145 PMC3458969

