# Supplementary figures and images for: Case Report: Hypocomplementemic urticarial vasculitis syndrome in a pediatric patient with complement factor 1 deficiency
Source: Front Pediatr. 2024 Sep 23;12:1448094. doi: 10.3389/fped.2024.1448094 (PMC11456532; doi:10.3389/fped.2024.1448094)

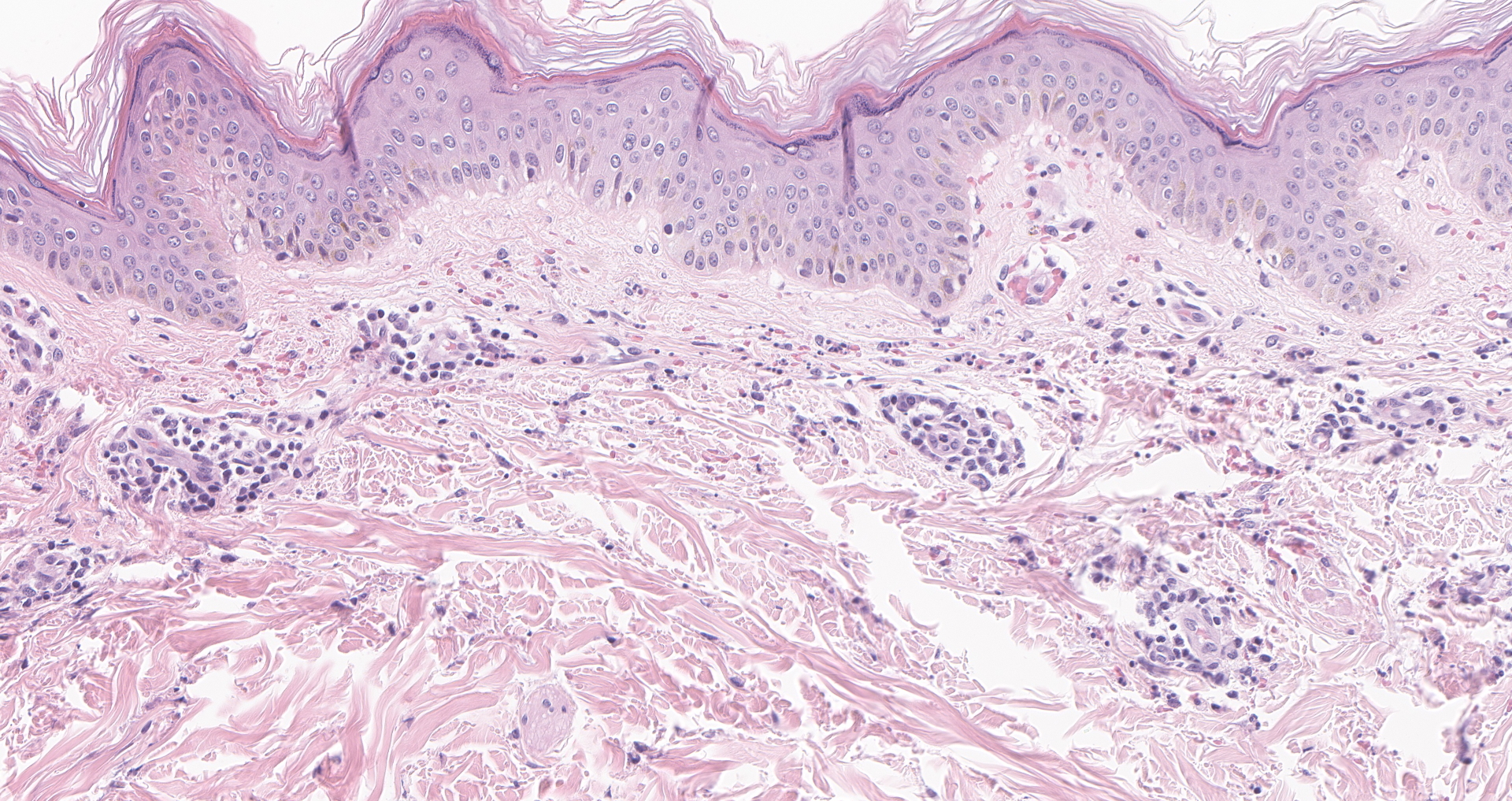

Supplement: Supplementary Figure 1 — Biopsy demonstrating leukocytoclastic vasculitis (200×). [file Image1.jpeg]

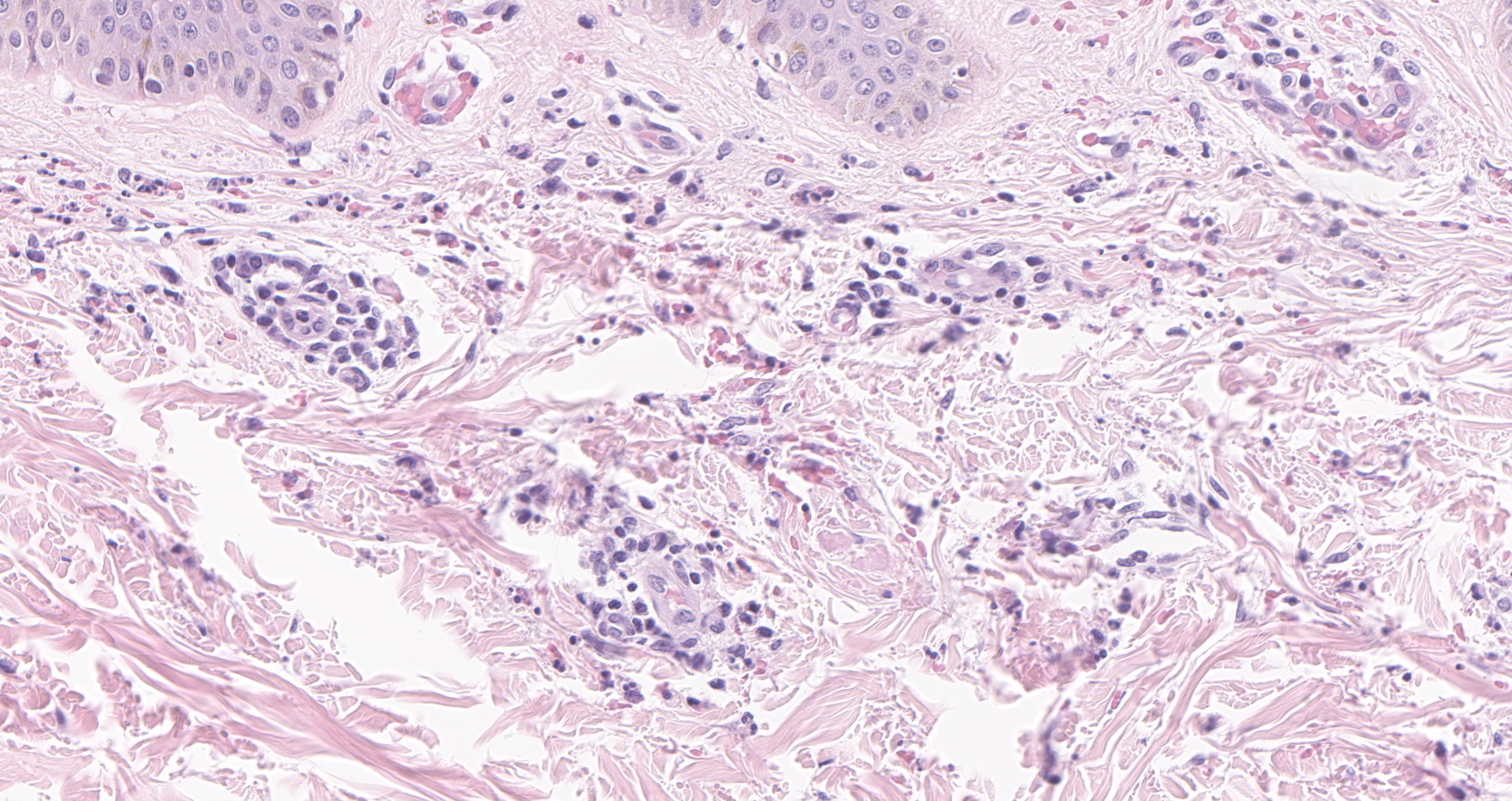

Supplement: Supplementary Figure 2 — Biopsy demonstrating leukocytoclastic vasculitis (300×). [file Image2.jpeg]
